# Co-Gradient Variation in Growth Rate and Development Time of a Broadly Distributed Butterfly

**DOI:** 10.1371/journal.pone.0095258

**Published:** 2014-04-17

**Authors:** Madeleine Barton, Paul Sunnucks, Melanie Norgate, Neil Murray, Michael Kearney

**Affiliations:** 1 Department of Zoology, The University of Melbourne, Parkville, Victoria, Australia; 2 School of Biological Sciences, Monash University, Clayton, Victoria, Australia; 3 Department of Genetics, La Trobe University, Melbourne, Victoria, Australia; Oxford Brookes University, United Kingdom

## Abstract

Widespread species often show geographic variation in thermally-sensitive traits, providing insight into how species respond to shifts in temperature through time. Such patterns may arise from phenotypic plasticity, genetic adaptation, or their interaction. In some cases, the effects of genotype and temperature may act together to reduce, or to exacerbate, phenotypic variation in fitness-related traits across varying thermal environments. We find evidence for such interactions in life-history traits of Heteronympha merope, a butterfly distributed across a broad latitudinal gradient in south-eastern Australia. We show that body size in this butterfly is negatively related to developmental temperature in the laboratory, in accordance with the temperature-size rule, but not in the field, despite very strong temperature gradients. A common garden experiment on larval thermal responses, spanning the environmental extremes of H. merope's distribution, revealed that butterflies from low latitude (warmer climate) populations have relatively fast intrinsic growth and development rates compared to those from cooler climates. These synergistic effects of genotype and temperature across the landscape (co-gradient variation) are likely to accentuate phenotypic variation in these traits, and this interaction must be accounted for when predicting how H. merope will respond to temperature change through time. These results highlight the importance of understanding how variation in life-history traits may arise in response to environmental change. Without this knowledge, we may fail to detect whether organisms are tracking environmental change, and if they are, whether it is by plasticity, adaptation or both.

## Introduction

Studies on broadly distributed insects have repeatedly shown geographic patterns in larval development time, growth rate and adult size in response to climatic gradients across the landscape [1], [2]. Geographic variation in these traits may arise in response to multiple selection pressures, involving phenotypic plasticity, genetic adaption, or both. Moreover, these life-history traits are often linked; changes in larval growth and development can have a direct impact on adult size [3], [4]. Exploring the mechanisms that give rise to geographic patterns in these traits is therefore complicated, but nonetheless required to understand how life histories change through space and time [5].

Latitudinal clines of insect traits form readily through phenotypically plastic responses to gradients in the environment. Bergmann's clines, for example, describe a positive correlation between latitude and body size [6], [7]. For insects in particular, this pattern has been attributed to the different thermal reaction norms of cell growth and differentiation – the ‘temperature-size rule’ [8]–[11]. According to this hypothesis, as body temperature rises, metabolic processes increase but the reaction norms are such that maturation increases proportionately more than growth, ultimately leading to a decrease in body size at maturity in response to increasing temperature.

Geographic variation in temperature seasonality, i.e. in the growing season, may also lead to patterns in insect size and development time across the landscape [12]–[15]. The duration of the growing season is usually negatively correlated with latitude, leading to ‘converse Bergmann’ clines in which body size increases towards the tropics [12], [16]. Variation in life-histories will also affect geographic patterns in insect physiology and size. Across climatic gradients, discrete shifts in voltinism, for example, may disrupt gradual shifts in larval physiology and lead to saw-tooth patterns in size [2], [14], [17].

Populations that encounter different conditions may undergo genetic adaptations in growth and development to ensure successful metamorphosis and to maximize adult fitness [18], [19]. Across the landscape, the effects of environment (temperature or seasonality) and genotype may act in a synergistic or antagonist manner on a given trait, either accentuating (co-gradient variation) or reducing (counter-gradient variation) phenotypic variation respectively [4], [20]–[23]. For example, direct larval development of a widespread European moth (*Eilema depressum*) occurs more readily at low latitudes due the effects of higher body temperature as well as underlying genotypic variation, an example of co-gradient variation [24]. Counter-gradient variation has been observed in patterns of growth rates in male larvae of the Speckled-wood butterfly (*Pararge aegeria*); high-latitude populations develop at an intrinsically faster rate to counteract the shorter, colder growing season [3]. If we were to compare phenotypic variation with corresponding thermal and seasonal gradients, while not accounting for genetic variation, for co-gradients we would likely over-estimate phenotypic plasticity in voltinism and growth rates, and under-estimate for counter-gradients. In extreme cases of counter-gradients, genetic adaptation may counteract fully the impacts of temperature on size or development time, resulting in no phenotypic variation across a species' range [25]. If the relatively easily measured traits of body size and adult emergence date are used as indicators of species' responses to environmental stress, species that do not appear to be responding may in fact be tracking changes through genetic adaptation [26]. With this in mind, studies that investigate the processes through which temperature-sensitive traits are maintained across different thermal environments may provide valuable insights into species' capacity to adapt to environmental change.

One species that exhibits little geographic variation in body size is the common brown butterfly (*Heteronympha merope*). The common brown is distributed across an extensive (17°) latitudinal range of eastern Australia, across which mean winter temperatures (larval developmental conditions) range from 7°C to 15°C. All populations are univoltine, with larvae feeding on native and introduced grass species. The strong thermal gradient across this species' distribution is reflected in geographic variation in adult emergence date [27]: adults at lower latitudes emerge earlier in spring. However, previous work [28] indicates a lack of geographic patterns in body size, confirmed here with additional data, three decades subsequently.

To investigate how body size is maintained in the field, we conducted a common garden study, testing the direct impact of developmental temperature on *H. merope*'s life history. By rearing larvae from wide-ranging populations under different temperatures in the laboratory, we were able to describe the impacts of rearing temperature on size, development time, and growth rate, and ascertain how these effects varied across the landscape.

## Materials and Methods

### Field collections

In 2008, butterflies were collected from 21 sampling locations throughout *Heteronympha merope*'s range (Table S1). Upon netting, adults were killed by treatment with acetone before being transported back to the laboratory. Thorax length was measured to 0.1 mm with digital callipers, classified (from the lateral view) as the distance from the dorso-anterior corner of the pro-thoracic segment to the centre of the posterior end of the meta-thorax. Using this protocol, the thoraces of a random selection of ten individuals were measured three times and analyzed with a nested analysis of variance model, the results of which confirmed that this measuring approach was accurate (only 7% of the total variation was due to differences between repeated measures of the same individual).

The average thorax length of butterflies within each population and sex was then examined in the light of latitude and climate variables (mean winter temperature, mean winter rainfall and pasture growth, see below for a description of these data) at each population using a linear regression model (with sex included as a fixed effect). Prior to performing the regression analyses, we confirmed that that there was no evidence of spatial autocorrelation in thorax length using both Mantel tests and the ‘Moran I’ value.

Butterflies were collected under permits issued by The Victorian Department of Sustainability and Environment (10004062), New South Wales Department of Environment and Climate Change (S12221), Forestry New South Wales (CO34155), South Australian Department for Environment and Heritage (K25349 2) and Queensland Parks and Wildlife Service (WITK04310307), and adheres to The University of Melbourne and Australian legal requirements for research on Lepidoptera that are not of conservation concern.

### A common garden experiment

**Figure 1:**
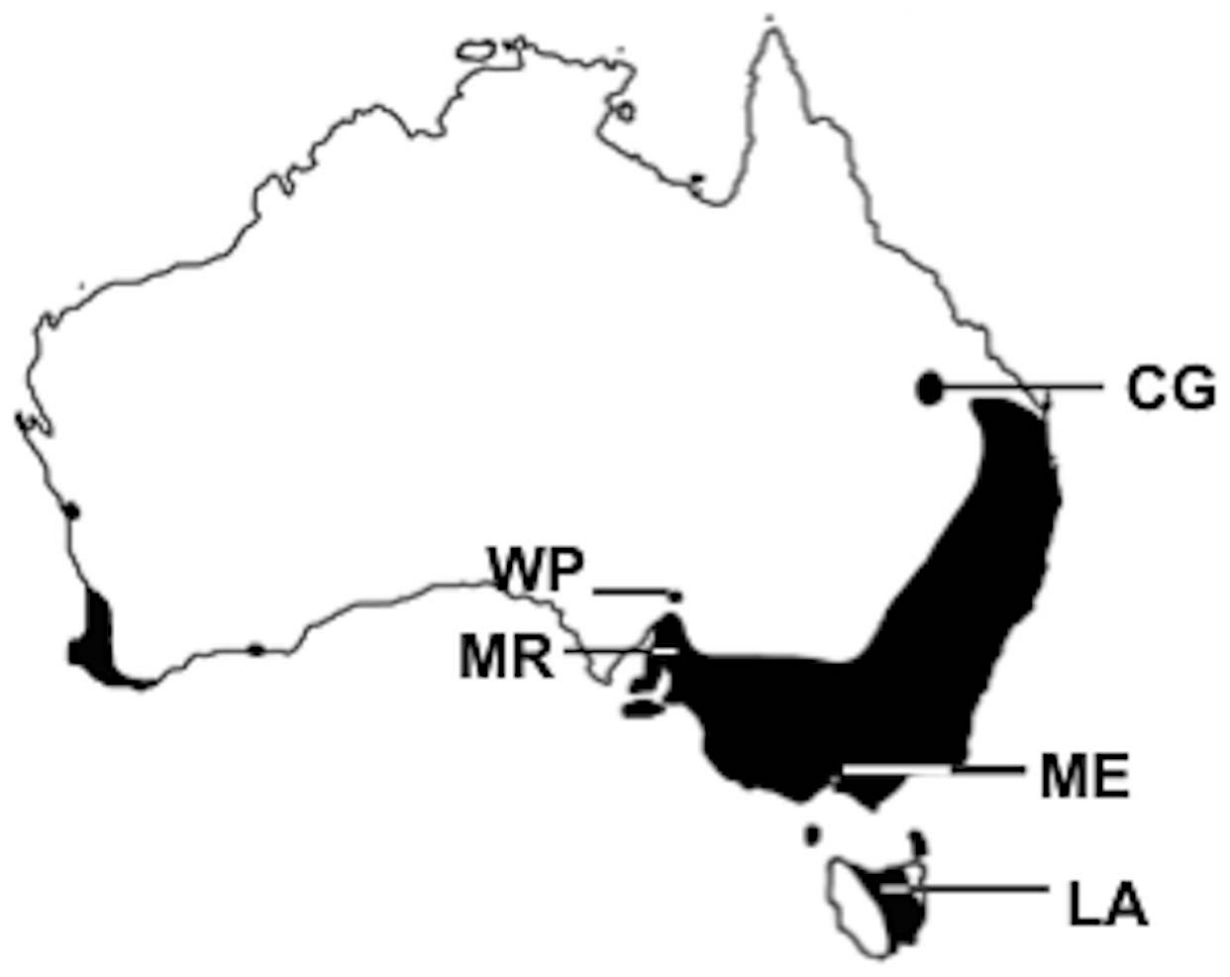
The distribution of *Heteronympha merope*. The distribution of *H. merope* across the Australian continent is indicated in black, and the location of collection sites of samples used in the common garden experiment are shown: Carnarvon Gorge (CG); Wilpena Pound (WP): Mt Remarkable (MR): Melbourne (ME); and Launceston (LA). Figure adapted from Norgate et al. [52].

For the rearing experiment, field-inseminated females were collected in March 2008 from five populations (Fig 1): Carnarvon Gorge (S 25.05°, E 148.23°; *n* = 3), Wilpena Pound (S 31.54°, E 138.59°; *n* = 3), Mt. Remarkable (S 32.92°, E 138.09°; *n* = 8), Melbourne (S 37.80°, E 145.33°; *n* = 6) and Launceston (S 41.45°, E 147.12°; *n* = 5). Once females were housed and ovipositing in the laboratory, eggs (from all populations) were collected daily and were stored at 12°C to ensure larval development of the five populations began simultaneously, maximizing comparability of the data from different populations. Upon hatching, larvae were weighed (Mettler Toledo XS205 Dual Range, >0.1 mg repeatability) and siblings divided into three constant temperature treatments: 8°C, 15°C or 20°C, with a 12∶12 light:dark photoperiod.

At least 10 offspring from each mother were allocated into each temperature treatment. Larvae were housed individually in cylindrical glass vials (height 130 mm, diameter 25 mm) closed with a moist foam stopper to maintain 100% humidity, and fed Panic velt grass (*Ehrharta erecta*) *ad libitum* (Pearse and Murray, 1982, Briscoe et al 2012).

We checked larvae daily for moulting, pupation and eclosion and recorded the number of days taken to complete each development phase. Pupae and adults were weighed, thorax length determined (methods as above), and forewing length and forewing area were measured using digital photographs and the program *Image J* (Wayne Rasband, National Institute of Health, USA). Growth rate was calculated according to the difference between the natural logarithms of pupal and hatchling weights, divided by the larval development time [3].

### Climatic data

Climatic data were extracted from historical continent-wide 0.05° (5 km) grids available through the Australian Water Availability Project (AWAP [29]). Average daily means of temperature and rainfall were calculated for the period of larval development (May-September). Estimates of monthly food availability (pasture growth, kg dry mass/ha) at each population's site were obtained from a process-based pasture model “AussieGRASS” [30] which is available as 0.05° grids (http://www.longpaddock.qld.gov.au/about/researchprojects/aussiegrass/index.html). The average monthly grass growth across the developmental period (May-September) at each site was calculated and used in analyses. Data from 2007 only, the winter preceding adult collection, were used in analyses of field collected specimens. To compare variation in physiological traits of laboratory-reared larvae with winter conditions of source location, long-term averages, from years spanning 1960–2007, were used.

### Statistical analysis

To test for variation in growth rates, development time and body size among groups in the laboratory reared specimens, measurements were analysed using three-way nested Analysis of Variance (ANOVA) models: temperature, sex and population were set as fixed effects with mother's identity nested within population as a random factor to capture part of the variation that may simply be inherent to the sibling groups. Such ‘brood effects’ described in this experiment encompass both genetic variation specific to each family group, as well as environmental variation experienced by the mothers in their source populations. Resources during larval development may be allocated differently to structural aspects (thorax length, wing shape and size) or reserves (body mass relative to length) depending on the sex, temperature and population of origin of the individual [31]. To ensure the variation in mass and size traits were consistent between sexes, populations and temperature treatments, we conducted an analysis of co-variance between adult and pupal mass, with thorax length, forewing area, and forewing length. To meet the assumptions of an ANOVA we log-transformed all data for size and development time prior to analyses. Pair-wise comparisons were performed using Tukey's HSD test.

The average measure of larval growth and development time for each population (for those in 15°C and 20°C, and for each sex) were subsequently analysed with respect to latitude, average winter temperature, average winter rainfall and pasture growth using linear regression models.

## Results

### Body size in the field

**Figure 2:**
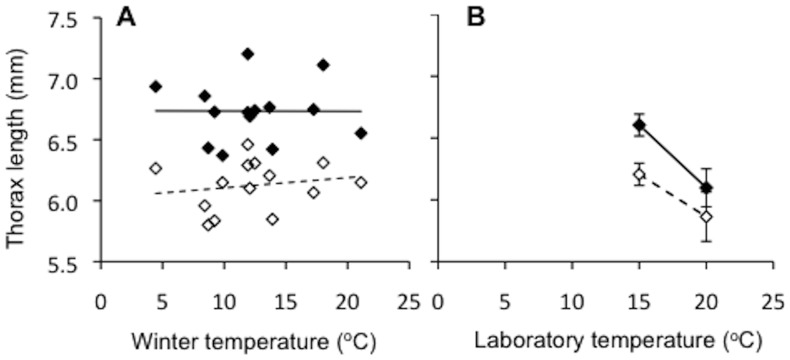
Discrepancies in thermal effects on body size. Average thorax length of (a) field-collected specimens with respect to average winter (May-September) temperature of source populations and (b) laboratory reared specimens, with respect to temperature treatment (Males  =  open diamonds; Females  =  closed diamonds). Error bars denote standard error of mean (SEM).

The thorax length of both male and female field-collected *Heteronympha merope* butterflies were uncorrelated with latitude, mean winter temperature, rainfall and pasture growth (Fig 2a, Table 1). Across all populations, the thorax length (which was significantly correlated with forewing length and area; Table S3) of male butterflies were on average smaller (61.1±0.25 mm) than their female counterparts (67.7±0.24 mm).

**Table 1:** An absence of geographic variation in body size.

	*df*	*MS*	*F*	*p*
Sex	1	3.62	70.26	**<0.001**
Latitude	1	0.07	1.32	0.26
Winter temperature	1	0.10	1.92	0.18
Winter rainfall	1	0.39	7.56	0.06
Pasture growth	1	0.01	0.11	0.75
Error	29	0.05		

Linear regression for the effects of sex, latitude, average winter (May-September) temperature, winter rainfall and winter pasture growth on the average thorax length of *Heteronympha merope* specimens from different collection sites. Degrees of freedom (df), Mean squares (MS), F and p values are shown.

### Temperature, sex, and larval growth and development

In the laboratory, sex and temperature had interactive impacts on larval development (Table 2); development time decreased with rising temperature, and female larvae required more days to develop than males at 8°C (+46 days) and at 15°C (+22 days) but not at 20°C. Male larvae grew faster than females in all conditions and, for both sexes; growth rate was positively correlated with temperature (Table 2).

**Table 2:** Impacts of temperature, sex and population on fitness traits.

		*df*	*MS*	*F*	*p*
Larval Development Time					
	Population	4,15	2.45	27.50	**<0.001**
	Female(pop)	15,103	0.14	1.085	0.38
	Temperature	2,103	0.67	276.40	**<0.001**
	Sex	1,103	0.02	75.40	**<0.001**
	Pop × Temp	8,103	0.01	1.80	0.09
	Pop × Sex	4,103	0.05	0.60	0.65
	Temp × Sex	2,103	0.01	6.10	**<0.01**
	Pop × Temp × Sex	8,103	0.01	1.00	0.48
	Error	103	1.06	0.01	
Larval Growth rate					
	Population	4,15	28.69	9.80	**<0.01**
	Female(pop)	15, 84	8.26	2.34	**<0.01**
	Temperature	2,84	8.35	104.92	**<0.001**
	Sex	1,84	0.53	30.33	**<0.001**
	Pop × Temp	8,84	0.40	1.80	0.09
	Pop × Sex	4,84	0.37	1.47	0.22
	Temp × Sex	2,84	0.31	1.75	0.18
	Pop × Temp × Sex	8,84	0.28	1.25	0.28
	Error	84	27.79	0.28	
Adult Mass					
	Population	4,15	157.70	0.71	0.60
	Female(pop)	15, 75	162.20	1.62	0.09
	Temperature	1,75	552.90	23.14	**<0.001**
	Sex	1,75	13.30	80.10	**<0.001**
	Pop × Temp	4,75	5.20	2.06	0.10
	Pop × Sex	4,75	17.10	0.76	0.55
	Temp × Sex	1,75	3.70	3.37	0.07
	Pop × Temp × Sex	4,75	7.40	0.47	0.76
	Error	75	662.00	7.40	

Summary statistics of the effects of temperature (Temp), sex, population (Pop), mother (female) and parameter interactions on larval development time, larval growth rate and adult mass of H. merope (three-way nested ANOVA). df, MS, F and p values are shown. Significant p values are given in bold.

In the 8°C treatment, no female adults successfully emerged, while only 8 male butterflies (of 17 individuals that pupated successfully) emerged but with abnormally large thoraces, mouth parts and legs, and with disproportionately small wings; these individuals survived for up to only three days. These responses to the 8°C treatment were evenly spread throughout the populations, suggesting that chronic exposure to 8°C during larval development is close to the lower thermal limit for *H. merope*. Due to such high mortality, individuals from this treatment were excluded from analyses of pupal development and adult size (see below). This is in keeping with the scope of the temperature-size rule, which applies only at benign temperatures [11].

The effect of temperature on pupal development time was nonetheless striking: pupae from the 20°C treatment developed in approximately half the time required by those in the 15°C treatment (F_1,86_ = 819.86; p<0.001). The difference in larval development time observed between the sexes persisted in the pupal phase, with females taking significantly longer to develop than males at both temperature treatments (F_1,86_ = 15.33; p<0.001).

There were also significant impacts of temperature and sex on body size traits. Pupae of both sexes were heaviest at 15°C, while the difference in pupal weight between the sexes increased with rising temperature; female pupae were 2%, 16% and 21% heavier than males in the 8°C, 15°C and 20°C treatments respectively (Sex×Temperature: *F*_2,94_ = 6.79; *p*<0.001). The significant interaction between sex and temperature on pupal mass was not detected however when data from the 8°C treatment were removed from the dataset (results not shown). Adults in the 15°C treatment were significantly heavier (Table 2) and had a longer thorax than those reared at 20°C (Fig 2b; *F*_1,34_ = 19.10; *p*<0.001) and, within each treatment, female butterflies were approximately 24% heavier than males (Table 2) and had a longer thorax (Fig 2b; *F*_1,34_ = 17.08; *p*<0.001). Thorax length, forewing area and forewing length were all strongly correlated with both pupal mass and adult mass. These significant patterns were consistent across all temperature, sex and population groups of laboratory reared specimens (Table S2).

### Geographic variation in larval growth and development

**Figure 3:**
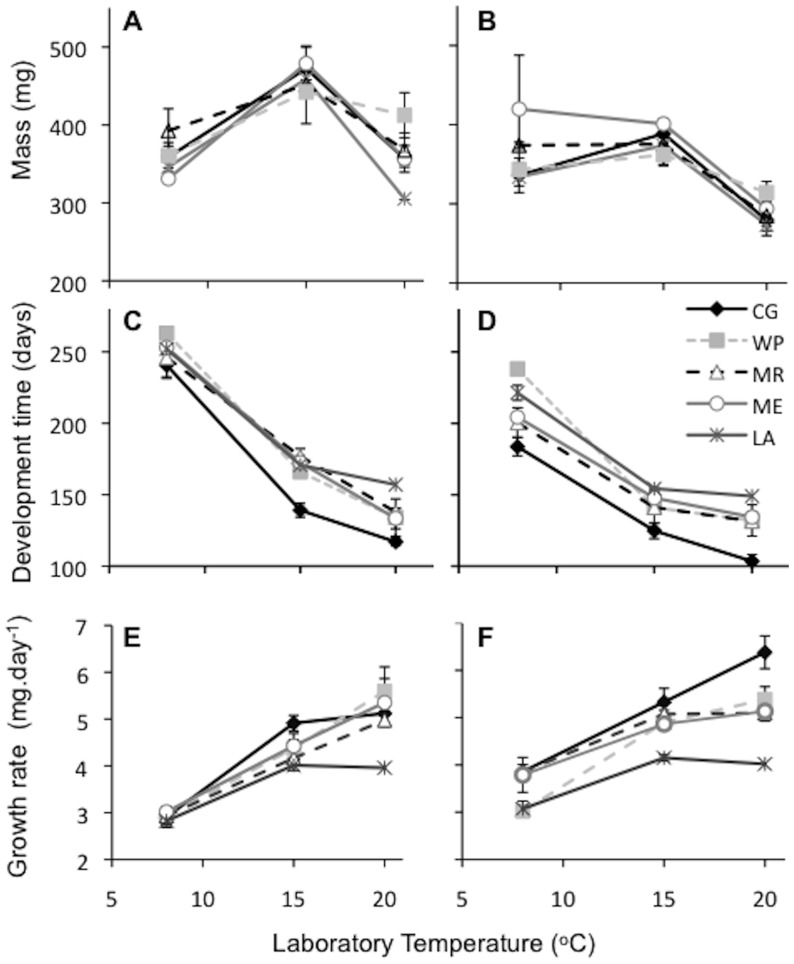
Geographic patterns in growth, development, but not size. No geographic variation in pupal mass (a,b) was detected, despite a significant population effect on (c,d) larval development and (e,f) growth rate of *H. merope* larvae reared in the laboratory. Data are sorted by sex, and populations are listed in order of decreasing latitude (north to south in the Southern hemisphere). Error bars denote SEM.

Population of origin had no effect on pupal mass, adult mass or thorax length of laboratory reared specimens (Table 3; Fig 3a,b). There was, however, a significant association between population and larval development time (Table 2; Fig 3c,d). Individuals from the site of lowest latitude, Carnarvon Gorge, required fewer days to develop than did the four higher-latitude populations. Furthermore, larvae from Carnarvon Gorge grew faster than those from Launceston, Melbourne and Mt Remarkable, while those from the highest-latitude site Launceston grew at a slower rate than those from Melbourne and Mt. Remarkable (Table 2; Fig 3e,f). There was also significant variation among family groups within the populations in the growth rate of female larvae at 20°C only (Table 2). Population also had significant impacts on pupal development time (*F*_4,15_ = 15.50; *p*<0.001), in a manner similar to that which was observed as larvae (described above).

**Table 3:** Latitudinal patterns in growth and development.

		Female				Male			
	T (°C)	R^2^	SE	t	p	R^2^	SE	t	p
Larval Development Time									
Latitude	15	0.582	0.8962	2.043	0.134	0.972	0.1708	10.120	**0.002**
Temperature		0.776	0.978	−3.225	**0.048**	0.985	0.1866	−13.910	**0.001**
Latitude	20	0.794	0.5988	3.400	**0.043**	0.890	0.5069	4.915	**0.016**
Temperature		0.781	0.02204	−3.266	**0.047**	0.970	0.3924	−9.889	**0.002**
Growth Rate									
Latitude	15	0.681	0.01865	−2.528	0.086	0.798	0.01912	−3.446	**0.041**
Temperature		0.799	0.9215	3.457	**0.041**	0.694	0.03512	2.609	0.080
Latitude	20	0.341	0.04971	−1.245	0.302	0.892	0.02698	−4.979	**0.016**
Temperature		0.181	0.0826	0.815	0.475	0.870	0.04423	4.471	**0.021**

Linear regressions of laboratory measured larval development time and growth rate of *H. merope* with respect to latitude and average winter temperature at source populations (n = 5). Results for each physiological trait are sorted by sex and laboratory rearing temperature (T). The R^2^, standard error (SE), t and p values are shown. Significant p-values are given in bold.

### Co-gradient variation in growth and development

**Figure 4:**
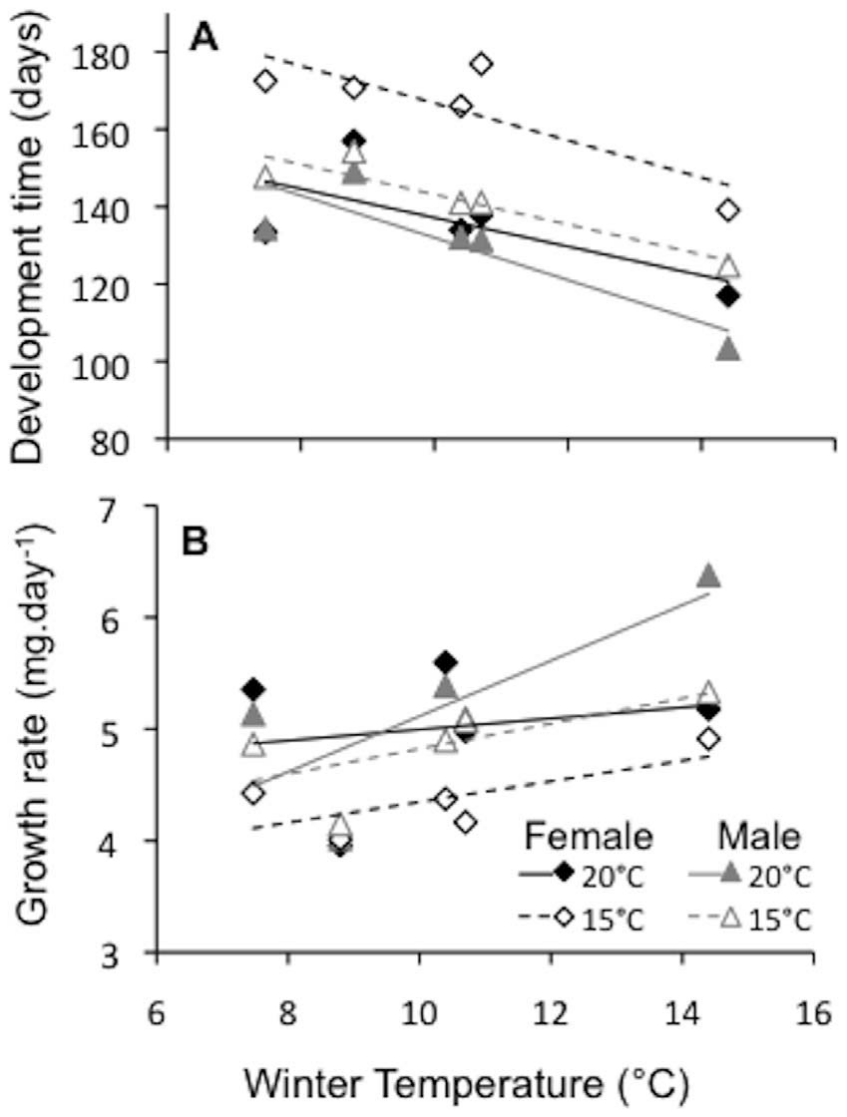
Adaptation of growth and development to local conditions. Regressions of laboratory measured (a) development and (b) growth rates of *H. merope* larvae with respect to average winter temperature (May – September) of source populations. Male and female data for the two temperature treatments are indicated.

Development time and growth rate of male larvae at both 15 and 20°C, and female larvae held at 20°C, were correlated with latitude and average winter temperature (Table 3). As average winter temperature of the source population increased, time spent in development declined, and larval growth rates increased (Fig. 4). There were no significant correlations between growth rate or development time with either average winter grass growth or winter rainfall (Table S4).

## Discussion

### Discrepancies in thermal effects on body size

Given that larvae of *Heteronympha merope* followed the well-established temperature-size rule in the laboratory (Fig 2a), we would expect to find a steep Bergmann's cline in body size across the common brown butterfly's range [9], [10]. However, no significant pattern in body size of field collected specimens was detected, with respect to latitude and climatic variables (Fig 2b). The absence of such a pattern is particularly surprising given that the common brown is distributed across a 17° latitudinal range, over which average winter temperature spans from 7°C to 15°C.The distribution of the genus *Heteronympha* is centered on relatively cool regions within Australia [27], which probably represents its ancestral environment. If this is true, as *H. merope*'s range has expanded into warmer climates it appears to have evolved mechanisms to buffer the impacts of temperature on size.

### Co-gradient variation across the landscape

The maintenance of a consistent body size across the broad distribution of *H.merope* may have arisen from variation in underlying physiological traits that directly affect adult size. Indeed, we found variation among sampling locations in larval growth rate and development time in the laboratory (Fig. 3). At all temperatures, individuals from the lowest-latitude population grew and developed faster than those from higher latitudes. Moreover, our findings suggest that variation in the environment (winter temperature) and genotype across *H. merope*'s broad distribution act synergistically on these larval traits. At low latitudes, in Carnarvon Gorge, we see warmer winter temperatures and intrinsically faster growth and development while at the other end of the distribution, low temperatures are coupled with relatively lower intrinsic growth and development rates. Consequently, if we were to rear specimens under their respective, local, field conditions, we may expect to see even greater variation in these traits [32].

Such co-gradient variation in growth rate alone may act to buffer the impacts of temperature on body size in the field. Although the warmer conditions at low latitudes lead to faster development (and thus a shorter growing period), a faster intrinsic growth rate may enable larvae to attain the same size as their southern (colder) counterparts. However, given development time also exhibits co-gradient variation across the landscape, the capacity to grow to a larger size at lower latitudes is likely to be reduced, as larvae have also evolved to develop at a faster rate in this region. It therefore remains unclear as to why the ‘temperature-size rule’ response that we observed in the laboratory is not, at least to some extent, expressed in the field.

### Trade-offs between growth, development and body size

The fact that the common brown's body size is so well buffered in the field despite the plastic effects of rearing temperature in the laboratory, suggests the existence of strong trade-offs between larval and adult fitness traits. This is perhaps not surprising as, theoretically, a large body size in animals generally confers greater fecundity and starvation resistance [33], and these expectations are met in several butterfly species [34], [35]. Moreover, such trade-offs are generally more pronounced under thermally stressful conditions [3]. Therefore, in populations of the common brown that encounter stressful temperatures throughout development, larvae may preferrentially allocate limited resources to attaining a threshold body size, at the expence of other life history triats not measured here.

Understanding how size is optimized at the expence of other life-history traits is by no means simple. Under laboratory conditions, there were no significant differences in survival across the five populations, for males or females, despite significantly more deaths occuring in the 8°C temperature treatment (Table S5). Moreover, body mass (used as an indication of body condition) was consistently, and significantly correlated with thorax length (an indication of structure) for all populations, sexes, and temperature treatments (Table S3). These consistent patterns suggest that life history traits associated with certain body allometries, such as flight capacity, are not indirectly compromised by suboptimal rearing temperatures [36].We were unable to measure the body mass of field collected specimens, however comparing mass to thorax length of butterflies sourced directly from the field may provide evidence for how the intensity of any potential trade-offs may vary across the common brown's range [31], [36]. Future studies that measure variation in flight capacity, fecundity and longevity across the common brown's range may also provide greater insight into the adaptive nature of the traits measured in this study.

### Alternative buffering opportunities

Across the common brown's range, variation in temperature experienced by larvae may to some degree be buffered by behavioural means. Highly active female butterflies have some capacity to modify the thermal environment of their offspring by altering the location of oviposition [37]–[39], and larvae may subsequently buffer temperature stress by selecting specific sites within their microhabitat in which to bask or feed [40], [41]. Differences in the timing of female oviposition across the landscape may similarly act to buffer variation in the thermal environments encountered by larvae at different sites, which would in turn reduce temperature effects on physiological traits and body size [37]. Indeed, female common brown butterflies in northern regions are known to lay eggs two to three weeks later than those at higher latitudes [42]. Consequently, at all sites across the common brown's range, larval thermal exposure may exceed a threshold number of degree-day units required to reach the standard body size.

### Seasonality as a potential stressor

Variation in larval growth and development may be associated with gradual changes in seasonality across the landscape [12], [14], [15], and if females do indeed vary the timing of oviposition between sites, growing season may play a significant role in shaping geographic patterns in larval physiology we observed in the laboratory [4]. In direct contrast to the majority of (northern hemisphere) Lepidoptera studied to date [2], [14], [20], [24], the duration of larval development in the cold-adapted common brown appears to be constrained at low, rather than high latitudes. A previous study conducted on the common brown demonstrated that final instar larvae were intolerant of constant temperatures over 20°C [37]. At low latitudes, where conditions become warmer earlier in spring, larvae may have evolved to grow and develop faster to ensure successful pupation prior to the onset of lethal conditions: larvae at Carnarvon Gorge experience a substantially shorter growing season, and thus, may have evolved to grow and develop at a faster rate. Seasonality and temperature may also affect the availability and quality of larval host plants, which would in turn affect variation in life history traits [43]. Systematic variation in the chemical composition of plant tissue, for example, may alter growth and development rates of the common brown, thereby over-riding the direct effects of temperature on final adult body size.

Temperature and seasonality co-vary with latitude, and so it is difficult to isolate their respective impact on adaptive physiological traits. Moreover, because all larvae were reared under the same photoperiod (used as a cue of seasonality [44]), quantifying the relative impacts of season length and temperature on physiology is beyond the scope of this study. Individuals in the field experience not only seasonal fluctuations in temperature, but also diurnal temperature variation - conditions that we did not simulate in the laboratory. These different thermal regimes may explain why variation in body size was observed in laboratory reared, but not field-collected, specimens [45], [46]. Further research that considers the effect of diurnal and seasonal temperature variation on *H. merope*'s life history would be informative, particularly as temperature (means and variances) and photoperiod are predicted to become de-coupled under future climate scenarios [47], [48].

### Broader implications

Rearing common brown specimens through multiple generations in the laboratory has proven unsuccessful, thus eggs were collected from field-inseminated rather than laboratory reared mothers. Consequently, the developmental and mating environments encountered by mothers at their site of origin, as well as the genetic variation among them, may have in turn led to the differences in larval physiology observed among populations [49], [50].The study design did not estimate between-population brood effects, and thus the interpretation of genetic differentiation must be made with some caution.

Nonetheless, our results suggest that both phenotypic plasticity and local adaptation may play a role in shaping *H. merope*'s responses to changes in temperature through time. Assuming that the common brown can adequately adapt to novel temperatures under climate change, co-gradient variation may produce more extensive changes in growth and development than would be expected based solely on a phenotypically plastic response to temperature change [24], [32]. In contrast, our finding of a consistent body size in wild populations of *H. merope* suggests that changes in body size in response to warming temperatures are likely to be smaller than predicted from laboratory thermal experiments.

While we have not fully succeeded in determining the mechanisms through which body size is maintained across the thermal gradient of *H. merope*, we have nonetheless highlighted the complex nature of species' responses to environmental variation. In general, the average body size of a number of organisms has declined in recent years, termed ‘the third universal response to climate change’ [51]. While the body size of a species may not respond to variation in temperature, our results indicate that they may, in fact, be tracking changes through underlying physiological, or behavioural, traits. Thus our current measures of declines in body size in response to temperature change [51] may under-estimate the extent of the physiological impacts of warming. A description of the processes through which species respond, or do not appear to respond, to environmental change, is thus required for a greater understanding of how climate constrains the survival of species.

## Supporting Information

Table S1 **Location of samples collected for analyses of body size in the field.** Latitude (°S) and Longitude (°E) are listed along with the number of samples of each sex that were considered and the average winter temperature (May-September) at each site.
(DOCX)

Table S2 **Adult and pupal mass were significantly correlated with thorax length, and adult mass with wing length and area, but these size correlations did not vary between the different temperature treatments, sexes or populations (as indicated by the non-significant interaction terms).**
(DOCX)

Table S3 **For field collected specimens, wing area and wing length were significantly correlated with thorax length for both male and female butterflies.**
(DOCX)

Table S4 **Statistical summary for linear regressions of laboratory measured larval development time and growth rate with respect to average winter rainfall and grass growth of source population.** Raw climate data were extracted from the “AussieGRASS” database (Carter, 2000), for years spanning (1960–2007). Results for each physiological trait are sorted by sex, and laboratory rearing temperature (T). No significant correlations were detected.
(DOCX)

Table S5 **Survivorship of laboratory reared individuals.** Results of a generalized linear model (with a quasibinomial error distribution, run using R version 3.0.1) indicate significantly higher rates of larval death in the 8°C treatment in comparison to both the 15°C and 20°C temperature treatments. All populations however, had similar death rates across all three temperature treatments (the interaction term was not significant, and thus removed from the analysis).
(DOCX)
