# Supplementary material for: Co-Gradient Variation in Growth Rate and Development Time of a Broadly Distributed Butterfly
Source: PLoS One. 2014 Apr 17;9(4):e95258. doi: 10.1371/journal.pone.0095258 (PMC3990641; doi:10.1371/journal.pone.0095258)
Supplement: Table S1 — Location of samples collected for analyses of body size in the field. Latitude (°S) and Longitude (°E) are listed along with the number of samples of each sex that were considered and the average winter temperature (May-September) at each site. (DOCX) [file pone.0095258.s001.docx]

**Table S1:** Location of samples collected for analyses of body size in the field. Latitude (°S) and Longitude (°E) are listed along with the number of samples of each sex that were considered and the average winter temperature (May-September) at each site.

|  | *Latitude* | *Longitude* | *Female* | *Male* | *Average Winter*  *Temperature* |
| --- | --- | --- | --- | --- | --- |
| Armidale | -30.49 | 151.64 | 4 | 7 | 12.07 |
| Carnarvon Gorge (CG) | -25.06 | 148.23 | 2 | 6 | 21.09 |
| Coonabarabran | -31.25 | 149.28 | 5 | 7 | 13.67 |
| Dubbo | -32.26 | 148.6 | 4 | 4 | 13.91 |
| Grenfell | -33.91 | 148.2 | 5 |  | 12.66 |
| Hobart | -42.56 | 147.36 | 5 |  | 8.84 |
| Honeysuckle Creek | -35.57 | 148.97 | 3 | 6 | 4.44 |
| Horsham | -36.71 | 142.3 | 4 |  | 10.93 |
| Launceston (LA) | -41.45 | 147.12 | 5 | 7 | 8.42 |
| Moruya Heads | -35.91 | 150.1 | 5 | 14 | 11.91 |
| Macedon | -37.42 | 144.56 | 5 |  | 14.21 |
| Myrtleford | -36.56 | 146.74 | 5 | 10 | 9.21 |
| Melbourne (ME) | -37.51 | 145.44 | 13 | 10 | 11.91 |
| Port Macquarie | -31.24 | 152.9 | 5 | 5 | 18.02 |
| Robe | -37.03 | 139.8 | 2 |  | 11.84 |
| Stirling | -35.02 | 138.71 | 5 |  | 11.42 |
| Toowoomba | -27.58 | 151.99 | 5 | 4 | 17.24 |
| Vittoria | -33.43 | 149.31 | 5 | 3 | 8.70 |
| Wentworth Falls | -33.73 | 150.39 | 3 | 10 | 12.47 |
| Wee Jasper | -35.12 | 148.67 | 4 | 8 | 9.86 |
| Wilpena Pound (WP) | -31.54 | 138.59 |  | 5 | 13.35 |
