# Supplementary material for: Co-Gradient Variation in Growth Rate and Development Time of a Broadly Distributed Butterfly
Source: PLoS One. 2014 Apr 17;9(4):e95258. doi: 10.1371/journal.pone.0095258 (PMC3990641; doi:10.1371/journal.pone.0095258)
Supplement: Table S2 — Adult and pupal mass were significantly correlated with thorax length, and adult mass with wing length and area, but these size correlations did not vary between the different temperature treatments, sexes or populations (as indicated by the non-significant interaction terms). (DOCX) [file pone.0095258.s002.docx]

**Table S2*:***Adult and pupal mass were significantly correlated with thorax length, and adult mass with wing length and area, but these size correlations did not vary between the different temperature treatments, sexes or populations (as indicated by the non-significant interaction terms).

|  |  | | ***df*** | ***MS*** | ***F*** | ***p*** |
| --- | --- | --- | --- | --- | --- | --- |
| Adult mass | |  |  |  |  |  |
|  | Thorax (length) | | 1 | 219274.29 | 31.70 | ***<0.001*** |
|  | Temperature | | 2 | 31130.68 | 4.50 | ***<0.05*** |
|  | Sex | | 1 | 227702.41 | 32.92 | ***<0.001*** |
|  | Population | | 4 | 6727.35 | 0.97 | 0.43 |
|  | Thorax × Temperature | | 2 | 7350.94 | 1.06 | 0.35 |
|  | Thorax × Sex | | 1 | 8455.57 | 1.22 | 0.27 |
|  | Thorax × Population | | 4 | 6170.86 | 0.89 | 0.48 |
|  | Error | | 51 | 6917.61 |  |  |
| Adult mass | |  |  |  |  |  |
|  | (Forewing) Area | | 1 | 219191.00 | 35.02 | ***<0.001*** |
|  | Temperature | | 2 | 26571.32 | 4.25 | ***<0.05*** |
|  | Sex | | 1 | 128693.09 | 20.56 | ***<0.001*** |
|  | Population | | 4 | 13414.35 | 2.14 | 0.09 |
|  | Area × Temperature | | 2 | 2076.53 | 0.33 | 0.72 |
|  | Area × Sex | | 1 | 185.01 | 0.03 | 0.86 |
|  | Area × Population | | 4 | 4873.23 | 0.78 | 0.54 |
|  | Error | | 46 | 6258.69 |  |  |
| Adult mass | | |  |  |  |  |
|  | (Forewing) Length | | 1 | 130814.24 | 21.47 | ***<0.001*** |
|  | Temperature | | 2 | 17283.72 | 2.84 | 0.07 |
|  | Sex | | 1 | 161154.91 | 26.45 | ***<0.001*** |
|  | Population | | 4 | 17604.05 | 2.89 | 0.06 |
|  | Length × Temperature | | 2 | 134.69 | 0.02 | 0.98 |
|  | Length × Sex | | 1 | 8993.24 | 1.48 | 0.23 |
|  | Length × Population | | 4 | 7083.46 | 1.16 | 0.34 |
|  | Error | | 42 | 6092.68 |  |  |
| Pupal mass | |  |  |  |  |  |
|  | Thorax (length) | | 1 | 1178844.90 | 54.46 | ***<0.001*** |
|  | Temperature | | 2 | 215315.48 | 9.95 | ***<0.001*** |
|  | Sex | | 2 | 274842.02 | 12.70 | ***<0.001*** |
|  | Population | | 4 | 5580.11 | 0.26 | 0.90 |
|  | Thorax × Temperature | | 2 | 41635.96 | 1.92 | 0.16 |
|  | Thorax × Sex | | 2 | 16196.46 | 0.75 | 0.48 |
|  | Thorax × Population | | 4 | 25710.75 | 1.19 | 0.33 |
|  | Error | | 58 | 21647.41 |  |  |
