# Supplementary material for: Co-Gradient Variation in Growth Rate and Development Time of a Broadly Distributed Butterfly
Source: PLoS One. 2014 Apr 17;9(4):e95258. doi: 10.1371/journal.pone.0095258 (PMC3990641; doi:10.1371/journal.pone.0095258)
Supplement: Table S3 — For field collected specimens, wing area and wing length were significantly correlated with thorax length for both male and female butterflies. (DOCX) [file pone.0095258.s003.docx]

**Table S3*:*** For field collected specimens, wing area and wing length were significantly correlated with thorax length for both male and female butterflies.

|  |  | | ***df*** | ***MS*** | ***F*** | ***p*** |
| --- | --- | --- | --- | --- | --- | --- |
| Female | |  |  |  |  |  |
|  | Wing Length | | 1 | 2.34 | 29.40 | ***<0.001*** |
|  | Error | | 94 | 0.08 |  |  |
|  |  | |  |  |  |  |
|  | Wing area | | 1 | 2.04 | 25.61 | ***<0.001*** |
|  | Error | | 98 | 0.08 |  |  |
|  |  | |  |  |  |  |
| Male | |  |  |  |  |  |
|  | Wing Length | | 1 | 1.92 | 29.25 | ***<0.001*** |
|  | Error | | 79 | 0.07 |  |  |
|  |  | |  |  |  |  |
|  | Wing area | | 1 | 0.00 | 0.00 | ***0.95*** |
|  | Error | | 77 | 0.08 |  |  |
