# Supplementary material for: Co-Gradient Variation in Growth Rate and Development Time of a Broadly Distributed Butterfly
Source: PLoS One. 2014 Apr 17;9(4):e95258. doi: 10.1371/journal.pone.0095258 (PMC3990641; doi:10.1371/journal.pone.0095258)
Supplement: Table S4 — Statistical summary for linear regressions of laboratory measured larval development time and growth rate with respect to average winter rainfall and grass growth of source population. Raw climate data were extracted from the “AussieGRASS” database (Carter, 2000), for years spanning (1960–2007). Results for each physiological trait are sorted by sex, and laboratory rearing temperature (T). No significant correlations were detected. (DOCX) [file pone.0095258.s004.docx]

**Table S4**: Statistical summary for linear regressions of laboratory measured larval development time and growth rate with respect to average winter rainfall and grass growth of source population. Raw climate data were extracted from the “AussieGRASS” database (Carter, 2000), for years spanning (1960-2007). Results for each physiological trait are sorted by sex, and laboratory rearing temperature (T). No significant correlations were detected.

|  |  |  | | | |  | Female | | | |  |  |  |  | | Male | |  | |  |
| --- | --- | --- | --- | --- | --- | --- | --- | --- | --- | --- | --- | --- | --- | --- | --- | --- | --- | --- | --- | --- |
|  |  | T (°C) | | | | R^2^ | | SE | t | p | | |  | R^2^ | SE | | t | | p | |
| ln(larval development) | |  | |  |  |  | |  |  |  | | |  |  |  | |  | |  | |
|  | Grass growth | | 15 | | | 0.014 | | 2.9523 | 0.206 | 0.850 | | |  | 0.225 | 1.913 | | 0.932 | | 0.420 | |
|  | Rainfall | |  | | | 0.533 | | 5.017 | 1.850 | 0.161 | | |  | 0.162 | 4.911 | | 0.760 | | 0.502 | |
|  |  | |  | | |  | |  |  |  | | |  |  |  | |  | |  | |
|  | Grass growth | | 20 | | | 0.041 | | 2.7709 | 0.359 | 0.743 | | |  | 0.095 | 3.112 | | 0.562 | | 0.614 | |
|  | Rainfall | |  | | | 0.242 | | 6.083 | 0.978 | 0.400 | | |  | 0.215 | 7.156 | | 0.907 | | 0.431 | |
| Growth rate | |  | |  |  |  | |  |  |  | | |  |  |  | |  | |  | |
|  | Grass growth | | 15 | | | 0.001 | | 0.07077 | -0.038 | 0.972 | | |  | 0.181 | 0.08267 | | -0.814 | | 0.475 | |
|  | Rainfall | |  | | | 0.494 | | 0.1243 | -1.711 | 0.186 | | |  | 0.029 | 0.22225 | | -0.298 | | 0.785 | |
|  |  | |  | | |  | |  |  |  | | |  |  |  | |  | |  | |
|  | Grass growth | | 20 | | | 0.030 | | 0.12931 | -0.306 | 0.779 | | |  | 0.094 | 0.16769 | | -0.557 | | 0.616 | |
|  | Rainfall | |  | | | 0.116 | | 0.3048 | -0.627 | 0.575 | | |  | 0.252 | 0.3761 | | -1.006 | | 0.389 | |
