# Supplementary material for: Co-Gradient Variation in Growth Rate and Development Time of a Broadly Distributed Butterfly
Source: PLoS One. 2014 Apr 17;9(4):e95258. doi: 10.1371/journal.pone.0095258 (PMC3990641; doi:10.1371/journal.pone.0095258)
Supplement: Table S5 — Survivorship of laboratory reared individuals. Results of a generalized linear model (with a quasibinomial error distribution, run using R version 3.0.1) indicate significantly higher rates of larval death in the 8°C treatment in comparison to both the 15°C and 20°C temperature treatments. All populations however, had similar death rates across all three temperature treatments (the interaction term was not significant, and thus removed from the analysis). (DOCX) [file pone.0095258.s005.docx]

**Table S5**: Survivorship of laboratory reared individuals. Results of a generalized linear model (with a quasibinomial error distribution, run using R version 3.0.1) indicate significantly higher rates of larval death in the 8°C treatment in comparison to both the 15°C and 20°C temperature treatments. All populations however, had similar death rates across all three temperature treatments (the interaction term was not significant, and thus removed from the analysis).

| *Survival rates of larvae* | *df* | *Deviance* | *Residual df* | *Residual Deviance* | *F* | *p* |
| --- | --- | --- | --- | --- | --- | --- |
| Population | 4 | 11.53 | 10 | 71.04 | 2.81 | 0.11 |
| Temperature | 2 | 61.65 | 8 | 9.39 | 30.02 | ***<0.001*** |
| Error |  |  | 14 | 82.57 |  |  |
